# Complete Genome Sequence of Novel Pseudorabies Virus Strain HNB Isolated in China

**DOI:** 10.1128/genomeA.01641-15

**Published:** 2016-01-28

**Authors:** Teng Yu, Fangzhou Chen, Xugang Ku, Yinxing Zhu, Hailong Ma, Subei Li, Qigai He

**Affiliations:** a State Key Laboratory of Agricultural Microbiology, College of Veterinary Medicine, Huazhong Agricultural University, Wuhan, China; b Animal Disease Diagnostic Center of Huazhong Agricultural University, Wuhan, China

## Abstract

The complete genome sequence of a novel pseudorabies virus, strain HNB, isolated from a dead weaned pig in China, was determined using next-generation sequencing. The viral genome sequence of HNB shared 90.6% nucleotide similarity with that of the traditional vaccine strain, the Bartha strain.

## GENOME ANNOUNCEMENT

Pseudorabies virus (PRV) is an enveloped double-stranded DNA virus that belongs to the family *Herpesviridae*, subfamily *Alphaherpesvirinae*, genus *Varicellovirus*, with a genome size of approximately 140 kb (1). The viral genome is similar to those of other alphaherpesviruses, consisting of a unique long (UL) region, internal repeat sequences (IRS), a unique short (US) region, and terminal repeat sequences (TRS) (2).

PRV is the causative agent of Aujeszky’s disease or pseudorabies (PR) in pigs (3). It can cause fatal infection in newborn pigs, severe respiratory signs in grower-finisher pigs, and reproductive failure in sows, causing great economic loss to the pig industry worldwide (4). The attenuated Bartha-K61 based vaccine (gE gene negative) and other live or killed vaccines have widely been used in China since the 1970s, and the eradication program is being conducted, thus porcine PR is well controlled worldwide including in China (5). However, since late 2011, many outbreaks of PR caused by novel PRV have occurred in major pig-producing provinces in China. It has been proven that the Bartha-K61 vaccine does not provide sufficient protection against novel PRV infection (5–7). In this study, a novel PRV strain, HNB, was isolated from brain tissue of dead weaned pigs from Bartha-K61-vaccinated pig farms. We determined the complete genome sequence of HNB using next-generation sequencing (NGS) technology on an Illumina MiSeq platform. The sequences were mapped to reference PRV complete genomes (GenBank accession no. NC_006151) and determined using the Velvet 1.2.08 and Gap Closer softwares. More than 92% of the genome sequence of strain HNB and 28 gaps were identified. These gaps in PRV strain HNB were further amplified by PCR for the purpose of sequencing analysis.

The complete genome of HNB was composed of 142,255 nucleotides (nt), with an average G+C content of 73.61%. The genomes contained a 101,078-nt UL region, a 16,134-nt IRS, an 8,909-nt US region, and a 16,134-nt TRS. The HNB strain contained 70 protein-coding genes (includes two copies of IE180, US1 genes, and major and minor forms of the US3 gene) and two latency-associated transcripts.

At the whole-genome level, HNB strain had 90.6%, 92.6%, and 91.7% identity with three non-Chinese PRV strains (Bartha, Kaplan, and Becker, with accession numbers JF797217 to JF797219, respectively), and 97.5%, 95.3%, 96.6%, and 96.0% identity with four Chinese PRV strains (TJ, ZJ01, HeN1, and JS-2012, with accession numbers KJ789182, KM061380, KP098534, and KP257591, respectively) isolated after 2012. Phylogenetic analysis of the complete genome sequence of the novel PRV strain HNB demonstrated that it was clustered in the same group as the four Chinese PRV strains, and a different group from the three non-Chinese PRV strains.

### Nucleotide sequence accession number.

The complete genome sequence of the PRV strain HNB has been deposited at GenBank with the accession number KM189914.
